# Damage-limited resolution for X-ray and electron microscopy of organic specimens

**DOI:** 10.1107/S2059798326002445

**Published:** 2026-04-10

**Authors:** Ray F. Egerton, Colin Nave

**Affiliations:** a Physics Department University of Alberta Edmonton ABT6G 2E1 Canada; b Diamond Light Source Harwell Science and Innovation Campus DidcotOX11 0DE United Kingdom; University of Oxford, United Kingdom

**Keywords:** radiation damage, dose-limited resolution, radiolysis, electron microscopy, X-ray microscopy

## Abstract

Analytical expressions for the damage-limited resolution (DLR) are developed and applied to X-ray and electron imaging of beam-sensitive specimens, allowing for variation of the characteristic radiation dose with spatial resolution. The dependence of DLR on specimen thickness is illustrated for the common modes of X-ray and transmission electron-microscope imaging. Similarities and differences between the radiolysis damage caused by electrons and X-rays are discussed. The meaning of a ‘Bragg boost’ in diffracted intensity is discussed.

## Abstract 2

Analytical expressions for the damage-limited resolution are developed and applied to X-ray and electron imaging of beam-sensitive specimens. The findings will guide future microscopy and instrument design.

## 1. Introduction

Radiation damage by radiolysis has long been recognized as a major problem for the imaging of organic and other beam-sensitive samples. The use of bright electron sources and improved electron optics (including the correction of lens aberrations) has made resolution in the transmission electron microscope (TEM) damage-limited for an increasingly wide range of samples. Radiolysis is also recognized as a major problem in X-ray microscopy, following the development of synchrotrons with improved beam brightness and tighter beam focusing.

The purpose of this article is to explore the connection between beam damage and image resolution in both forms of microscopy, and to compare the characteristics of electron and X-ray radiolysis with a view to obtaining further insight into the damage process.

## 2. Dose-limited resolution

Since electrons and X-rays are detected as particles, any recorded signal is quantized, resulting in a significant shot-noise component when the recorded intensity is low. In microscopy, this situation arises when trying to detect or analyze a small feature within a specimen, and leads to the concept of a dose-limited resolution.

**Figure 1:**
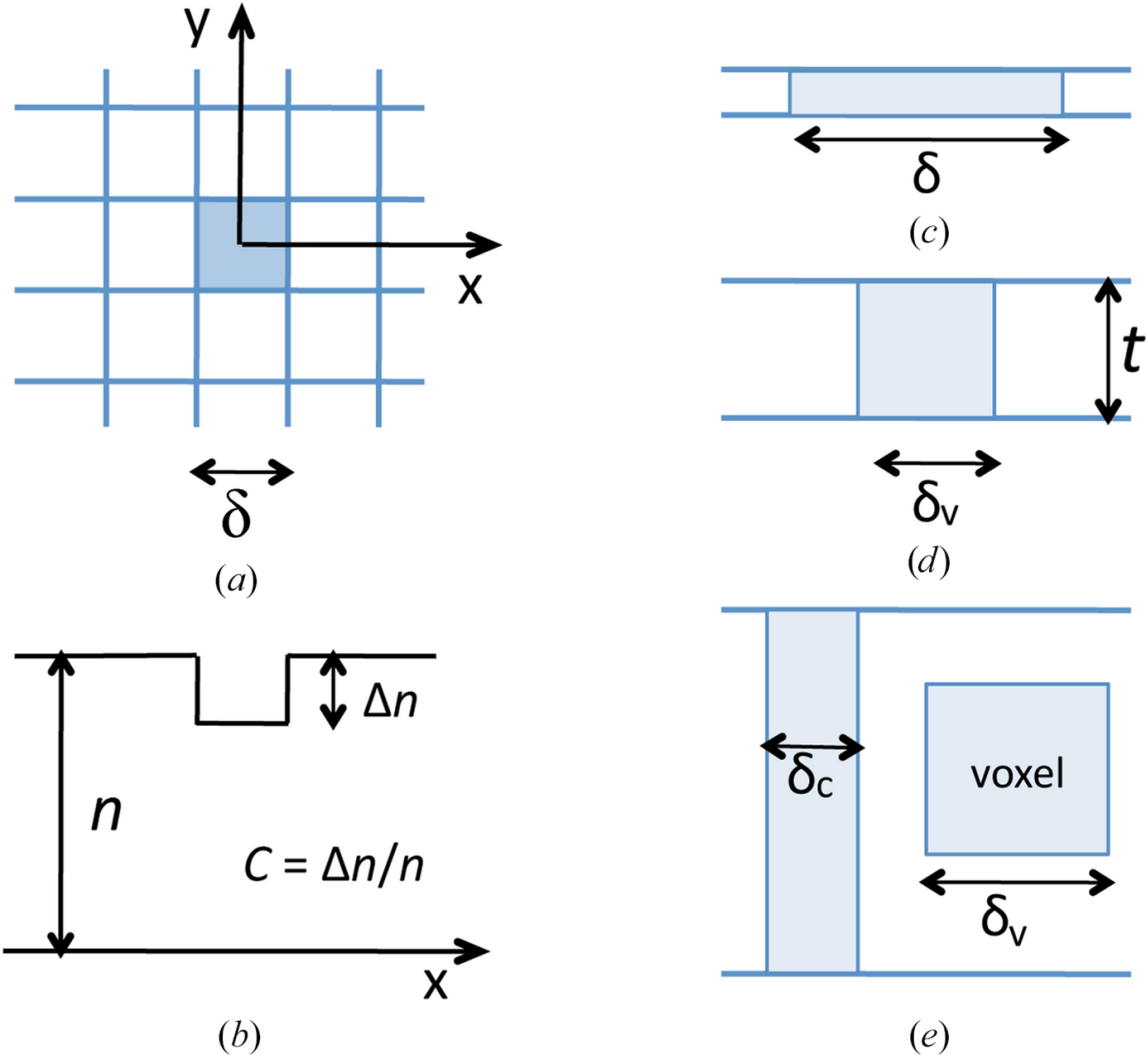
(*a*) Plan view of a specimen containing a square imaged feature of size δ surrounded by picture elements (pixels) of equal size. The analysis of round or spherical features will lead to identical equations, except for internal factors of π/4 or π/6. (*b*) Number of detected electrons per pixel as a function of distance *x* along the specimen. (*c*) Cross section of a very thin sample, showing the volume contributing to the damage-limited resolution (DLR) of dimension δ. (*d*) Cross section of a sample whose thickness *t* is *equal* to the size of the voxel DLR δ_v_. (*e*) Cross section or a thicker sample (*t* > δ_v_) showing a columnar DLR δ_c_ (appropriate to features extending between both surfaces) and a voxel DLR δ_v_ appropriate to equiaxed features.

Fig. 1 ▸(*a*) shows a small feature of size δ within the two-dimensional image of a thin specimen surrounded by ‘background pixels’ of equal size. If the feature extends between the top and bottom surfaces of the specimen, as in Fig. 1 ▸(*c*), the number of particles it contributes to the specimen image is
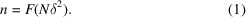
Here, *F* is a particle-collection efficiency and *N* is the particle fluence used to record the image: the flux in particles per unit area multiplied by the recording time. Fig. 1 ▸(*b*) is an intensity scan through the image, showing that (relative to its surroundings) the feature generates a ‘signal’ containing Δ*n* detected particles, given by
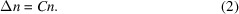
Here, *C* is the Weber contrast ratio of the feature (defined in Fig. 1 ▸*b*), which can be positive or negative depending on the imaging mode and scattering or absorptive power of the feature.

According to Poisson statistics, the shot noise associated with this signal is *n*^1/2^, resulting in a signal-to-noise ratio (SNR) given by
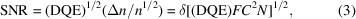
where DQE is the detective quantum efficiency of the imaging system. The value of δ that corresponds to a barely adequate SNR is referred to as a dose-limited resolution.

If the particle flux is weak, a long recording time is required to achieve an adequate SNR and the resolution becomes dose-limited, which is certainly the case for neutron beams (Henderson, 1995 ▸) and has previously been true for hard X-rays (Rez, 2021 ▸). With modern X-ray synchrotrons and electron microscopes, the fluence *N* that can be used is more likely to be limited by radiation damage, leading to a *damage-limited* resolution (DLR).

**Figure 2:**
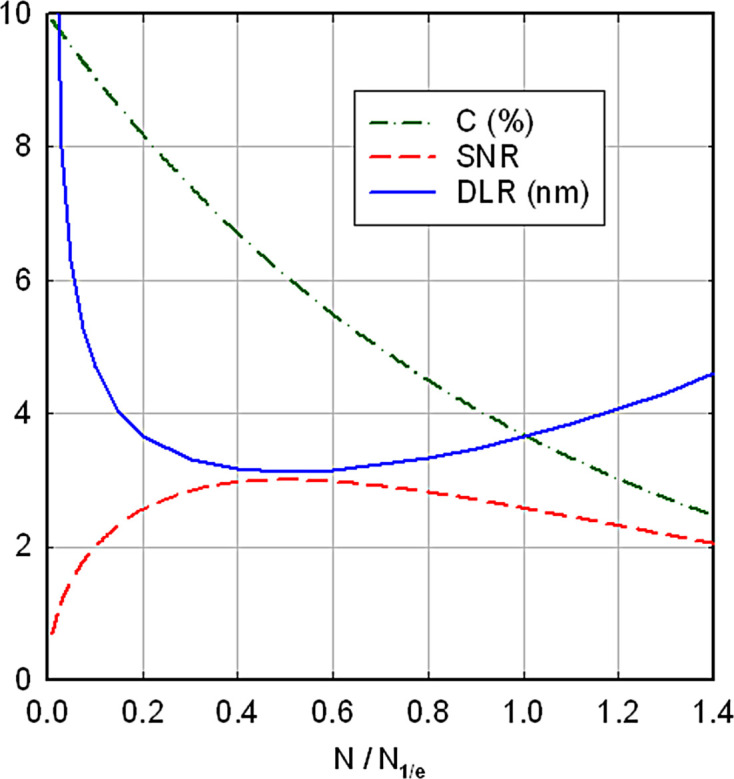
Dashed–dotted green curve: contrast ratio *C* given by equation (4) with *C*_0_ = 10%. Dashed red curve: the resulting signal-to-noise ratio given by equation (3) with δ = 3.13 nm, DQE = 0.5, *F* = 0.1 and *N*_1/e_ = 10^4^ nm^−2^. Blue curve: the corresponding dose-limited resolution for SNR = 3, given by equation (6). All data are plotted as a function of particle fluence *N* divided by the characteristic fluence *N*_1/e_.

In beam-sensitive materials, the predominant damage process is radiolysis (ionization damage). For electrically conducting samples (including metals) radiolysis is absent, leaving knock-on displacement as the damage mechanism above the knock-on threshold energy. However, this process is slow: for organic materials, radiolysis provides scattering and stopping-power cross sections that are several orders or magnitude larger (Egerton, 2012 ▸). Radiolysis destroys the original specimen structure and causes a loss of image contrast. Electron and X-ray measurements show that the fluence dependence of the contrast is often approximately exponential (see, for example, Glaeser & Taylor, 1978 ▸; Owen *et al.*, 2006 ▸; de la Mora *et al.*, 2020 ▸; Egerton, 2019 ▸), so that the Weber contrast ratio of the feature is
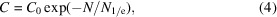
where *C*_0_ is the contrast at minimal exposure and *N*_1/e_ is a characteristic or critical fluence (a factor of 1.44 larger than the fluence *N*_1/2_ at which *C* falls by a factor of 2). As a result, the signal-to-noise ratio reaches a maximum value at a fluence *N* = *N*_1/e_/2, as shown in Fig. 2 ▸. This condition defines an *optimum exposure*, corresponding to a signal-to-noise ratio given by
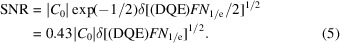
Inverting equation (5), the value of δ (the size of the smallest detectable feature, as limited by radiation damage) is given by
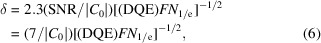
if we take SNR = 3 as being sufficient for signal detection with reasonable certainty (Hawkes & Spence, 2007 ▸). Sometimes SNR = 5 is used, based on trials in which people identified image features on a TV screen (Rose, 1948 ▸).

## 3. Thickness dependence of the DLR

When the specimen is very thin, the transmission image represents a cross section through its internal structure. As depicted in Fig. 1 ▸(*c*), the smallest detectable volume extends throughout the sample thickness and constitutes a *column* whose height is equal to the specimen thickness (*t*). The image contrast (if not too large) can then be written as |*C*_0_| = *t*Δ*k*, where Δ*k* is the contrast per unit depth of sample, due to a difference in scattering or absorption power between the feature and its surroundings. The column DLR is then
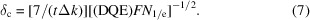
As the specimen thickness is increased, the contrast and SNR increase, providing an improvement in DLR due to the larger volume of specimen giving rise to the signal. When the damage-limited resolution becomes equal to the specimen thickness, the smallest detectable volume (SDV) is equiaxed (cubic in Fig. 1 ▸*d*). A further increase in thickness could provide even better resolution if the height of the SDV exceeds its width (Fig. 1 ▸*e*), a situation that only applies to specimen features (such as grain boundaries or cell walls) that run from the top to the bottom of the specimen and lie roughly parallel to the imaging beam.

More typically, specimen features are equiaxed (roughly cubic or spherical) and are represented by a cubic-voxel SDV (Fig. 1 ▸*e*). The damage-limited resolution is still given by equation (6) but with the image contrast reduced to |*C*_0_| = (*δ/t*)(*t*Δ*k*) = (δ)(Δ*k*), so that the *voxel* DLR is given by
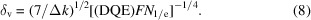


In agreement with Howells *et al.* (2009 ▸), this voxel resolution is proportional to the one-quarter power of the particle fluence; it remains largely independent of thickness until plural-scattering or absorption effects reduce the signal efficiency *F* and degrade the DLR.

## 4. Variation of the characteristic fluence with spatial resolution

The characteristic fluence (*N*_1/e_) is often taken to be a property of the specimen material, for a given energy of the irradiating particles. However, irradiation causes the outer spots in an X-ray or electron-diffraction pattern (corresponding to higher resolution, *i.e.* smaller *d*-spacing) to fade more rapidly than the inner spots. Therefore, the value of *N*_1/e_ depends on the resolution associated with the recorded data.

**Figure 3:**
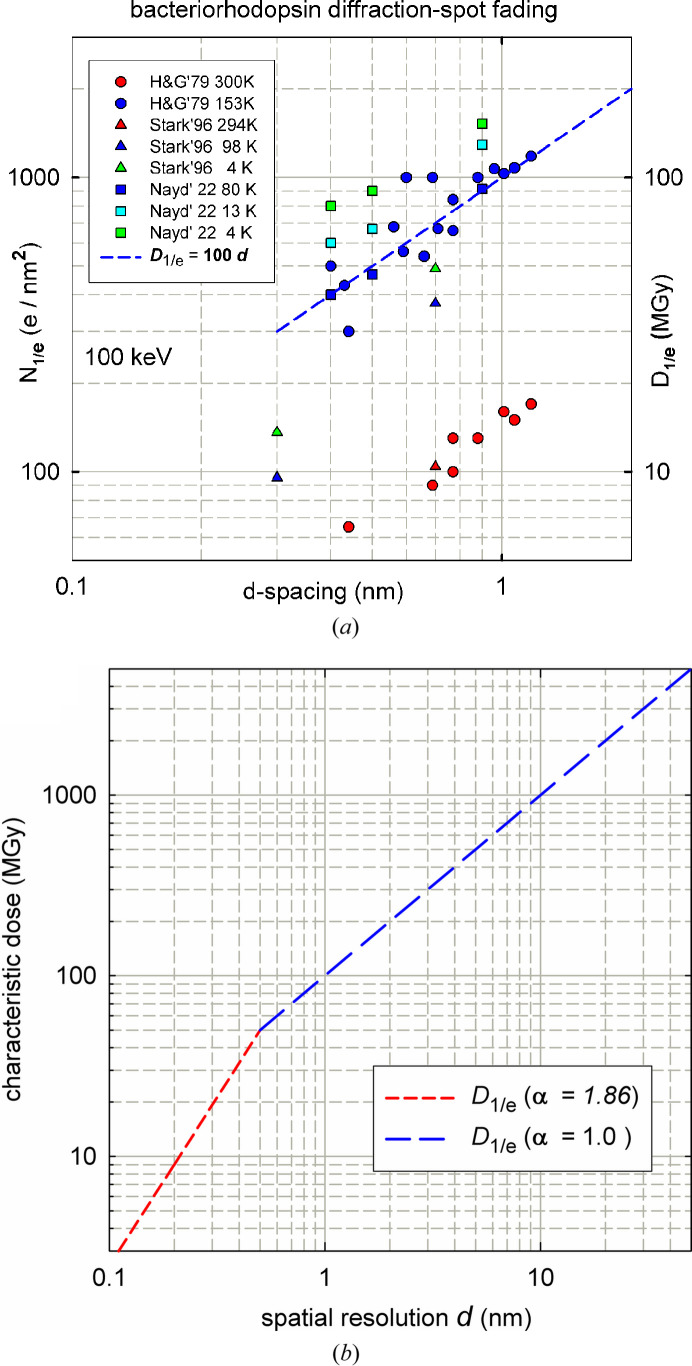
(*a*) Characteristic electron fluence (scaled for 100 keV electrons) as a function of the interplanar *d*-spacing, measured for bacteriorhodopsin by several authors (and at different temperatures) from the fading of diffraction spots. The right-hand scale shows the approximate characteristic dose in MGy. (*b*) Characteristic dose for several biomolecular crystals as a function of *d*-spacing, according to Atakisi *et al.* (2019 ▸), where α is defined by equation (12).

TEM measurements on bacteriorhodopsin at various temperatures, shown in Fig. 3 ▸(*a*), give this dependence as approximately linear, in agreement with X-ray data recorded for larger lattice-plane spacings by Howells *et al.* (2009 ▸). Taking *d* to be equal to the damage-limited resolution δ, the most appropriate characteristic fluence to use in equations (6), (7) and (8) is then
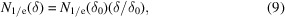
where *N*_1/e_(δ_0_) is the characteristic fluence that corresponds to a resolution δ_0_. Combining equation (9) with equations (7) and (8) gives
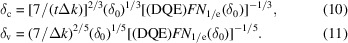


For resolutions better than 0.5 nm, X-ray data (Teng & Moffat, 2000 ▸; Sliz *et al.*, 2003 ▸; Bourenkov & Popov, 2010 ▸; Warkentin & Thorne, 2010 ▸; Owen *et al.*, 2014 ▸; Liebschner *et al.*, 2015 ▸; Nave *et al.*, 2016 ▸; Coughlan *et al.*, 2017 ▸) suggest that the dependence might be better represented by
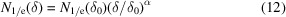
with α = 1.7 or 1.86 (Atakisi *et al.*, 2019 ▸). So, more generally, DLR values are given by
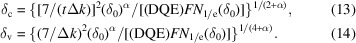


Damage to a crystal on a few-ångström scale can be thought of in terms of atom displacement from Bragg planes, for which kinematic diffraction theory suggests α = 2. There seems to be no published explanation for the value (α = 1) observed for lower resolution data, where damage is likely to involve secondary processes such as chemical reaction and diffusion. At long length scales, mass transport within an irradiated region might suggest that α should fall to 0.7, reflecting a *d*^1/2^ diffusion distance.

In relation to Fig. 3 of Howells *et al.* (2009 ▸), δ_v_ corresponds to the intersection of the line representing the *required* dose for imaging and a band of experimental data that represents the *maximum tolerable* dose. The tolerable dose used in our DLR calculations is that of Howells *et al.* (2009 ▸), based on measurements of diffraction-spot fading and consistent with more recent studies (Kmetko *et al.*, 2006 ▸; Owen *et al.*, 2006 ▸). This characteristic dose is shown by the blue curve in Fig. 3 ▸(*b*) and is given by *D* (MGy) = 100*d* (nm).

## 5. Conversion of fluence to radiation dose

Before applying the above equations to X-ray and electron imaging, we discuss the use of gray units for radiation dose. Radiolysis begins with energy transfer, and one gray (Gy) corresponds to the deposition of one joule per kilogram of sample. Lethal human exposure corresponds to a few tens of grays (60 Gy for glioblastoma delivered at 2 Gy per day for 30 days), but a more appropriate unit for electron and X-ray microscopy is the megagray (MGy).

For a thin specimen, in which only a small fraction of the photons is absorbed, the X-ray dose is given by
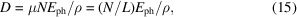
where μ is a linear absorption coefficient, *L* is an X-ray absorption length, *E*_ph_ is the photon energy and ρ is the physical density of the sample. In convenient units,
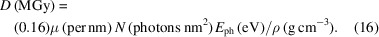
For situations in which the beam profile (X-ray flux density) is non-uniform, or the sample thickness is not small compared with *L*, an effective dose can be calculated using the *RADDOSE*-3*D* program (Zeldin *et al.*, 2013 ▸; Dickerson *et al.*, 2024 ▸).

For the case of electron irradiation, the dose in grays is
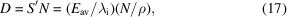
where *S*′ is the electron stopping power per unit mass of sample, *E*_av_ is the mean energy loss per inelastic collision and λ_i_ is the mean free path for inelastic scattering. In practical units,
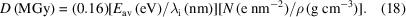


Conversion factors (Table 1 ▸) based on this formula are given in Egerton (2021 ▸), where the case of Gaussian focused probes is also discussed. Alternatively, values of *S*′ (in units of MeV cm^2^ g^−1^) for elements and common materials (and electron energies from 10 keV to 1 GeV) can be obtained from the *ESTAR* database (Berger *et al.*, 2005 ▸). The conversion is then
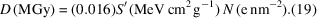


Of course, equations (15)–(19) can also be used to convert values of characteristic fluence to characteristic (or critical) dose *D*_1/e_, which forms a more convenient measure of the (inverse) radiation sensitivity of a material that is independent of electron or photon energy.

## 6. Numerical values for damage-limited resolution

As a practical example, and to provide comparison with previous publications, we concentrate on a single system: a cryocooled sample containing regions of a protein (composition H_50_C_30_N_9_O_10_S) surrounded by vitreous ice. Two questions are addressed: what is the smallest protein region that can be identified, and what resolution is possible within the protein? The latter situation might correspond to organelles within a biological cell, for which we have assumed a density difference of 10%. Some organelles differ by more than this amount, but the density variation within an organelle is likely to be less, so our DLR values could be optimistic.

**Figure 4:**
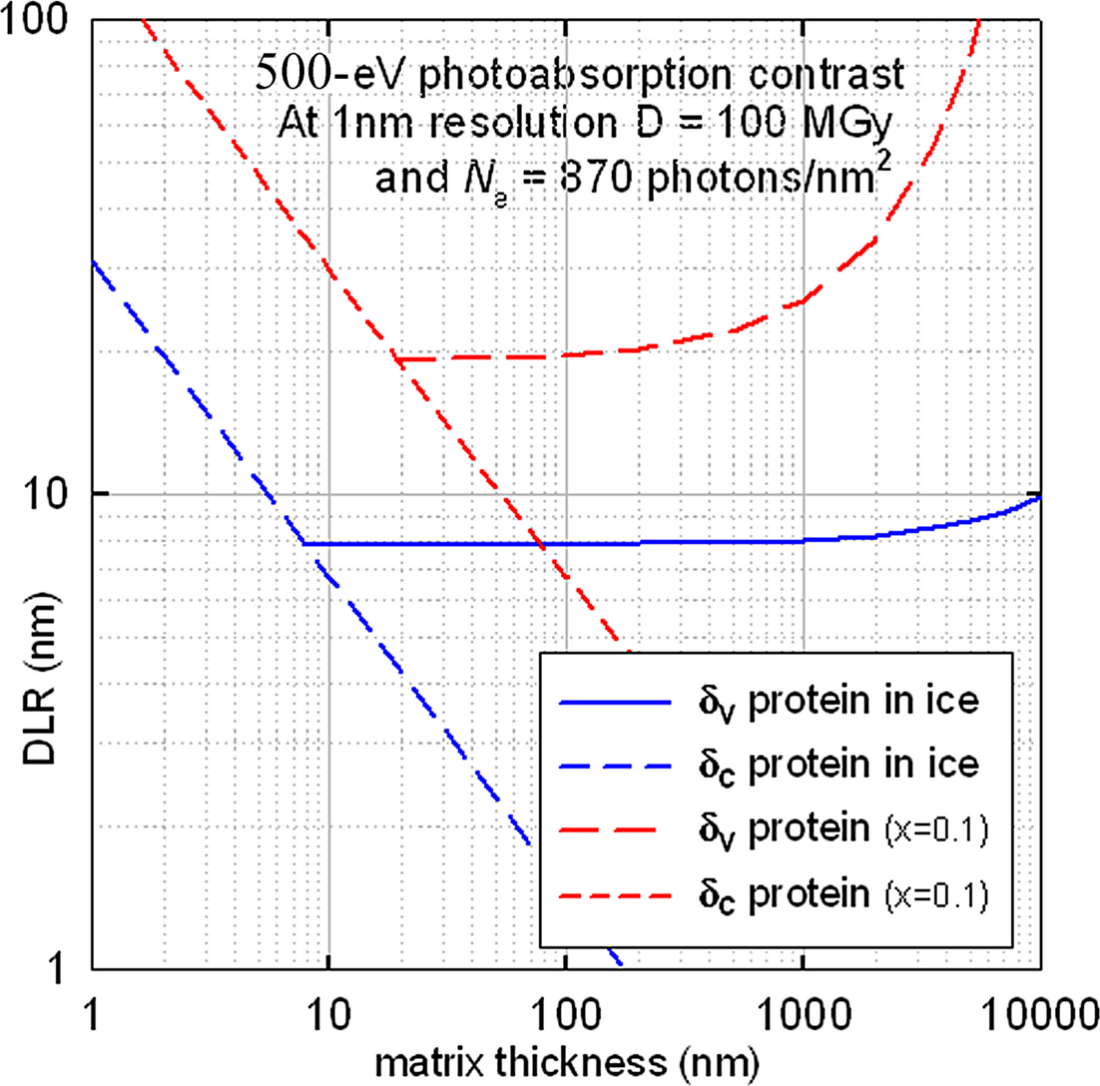
Damage-limited resolution for 500 eV X-ray absorption contrast between protein and ice (blue lines) and between proteins having a 10% difference in absorption coefficient (red lines). The X-ray dose *D* required for each resolution δ (expressed in nanometres) is *D* = (100δ) MGy.

**Figure 5:**
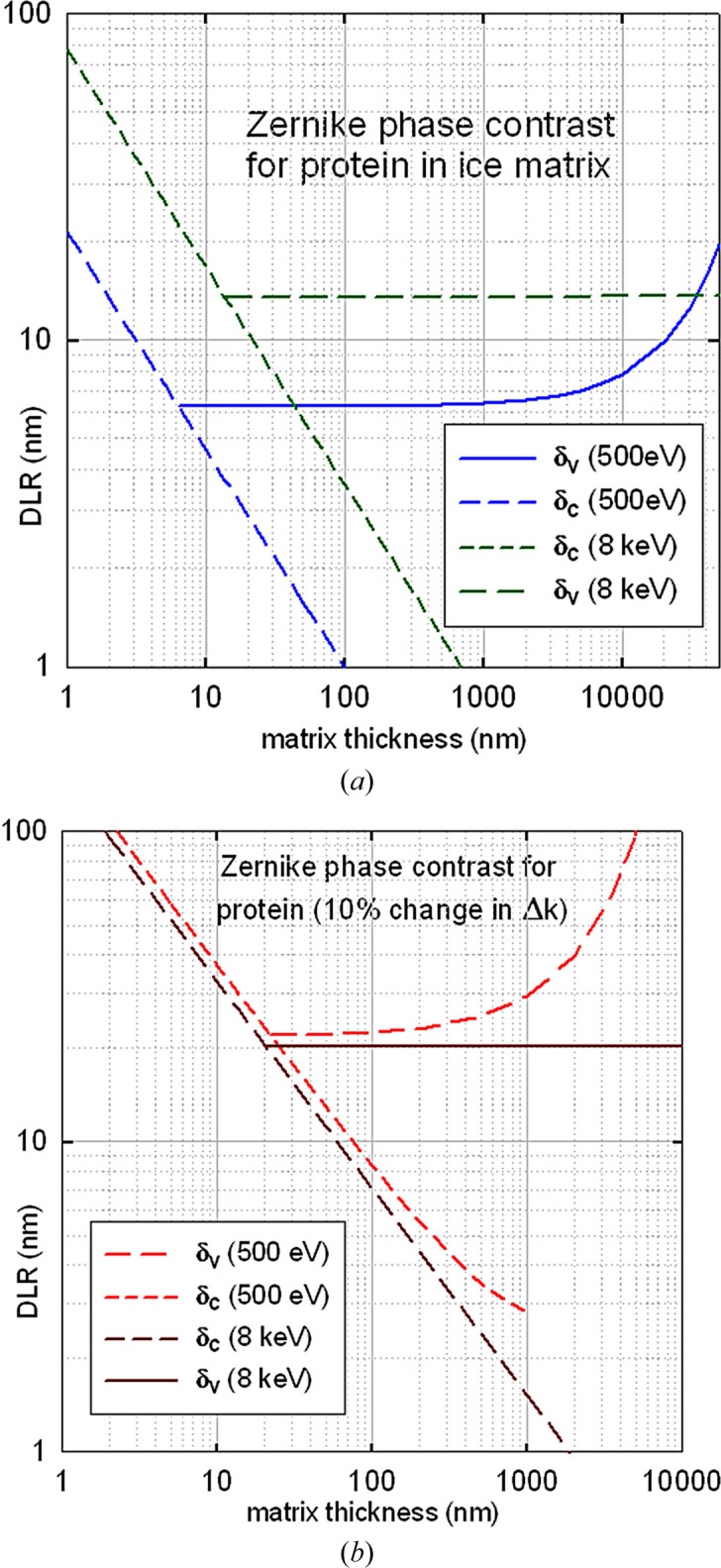
Calculated DLR for phase-contrast X-ray imaging of (*a*) protein in ice and (*b*) protein with a 10% difference in (1 + ɛ_1_). The X-ray dose *D* required for each resolution δ (expressed in nanometres) is *D* = (100δ) MGy.

**Figure 6:**
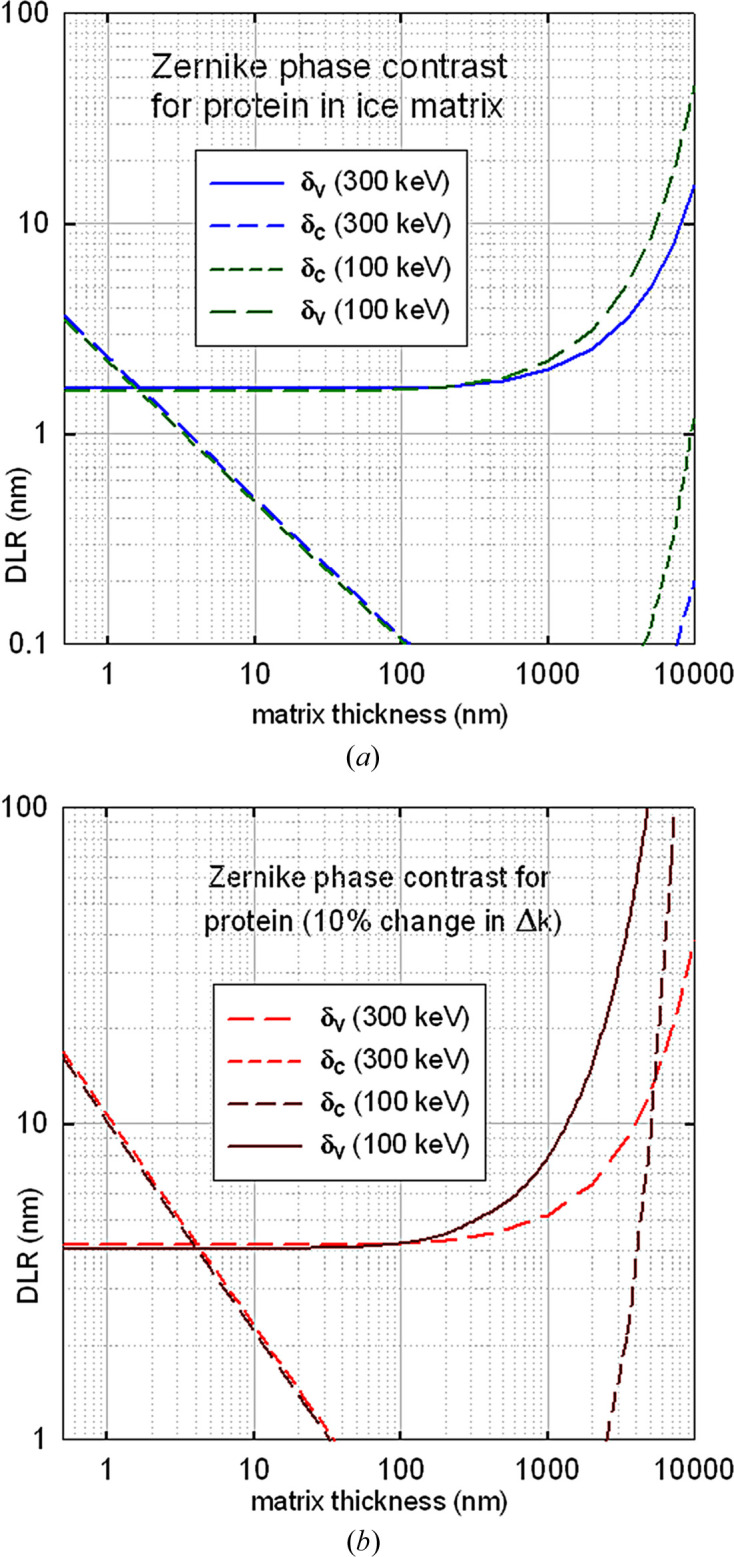
Voxel and column DLR for TEM phase contrast of (*a*) a protein feature embedded in ice and (*b*) protein with a 10% difference in MIP embedded in a protein matrix for electron energies of 300 and 100 keV.

**Figure 7:**
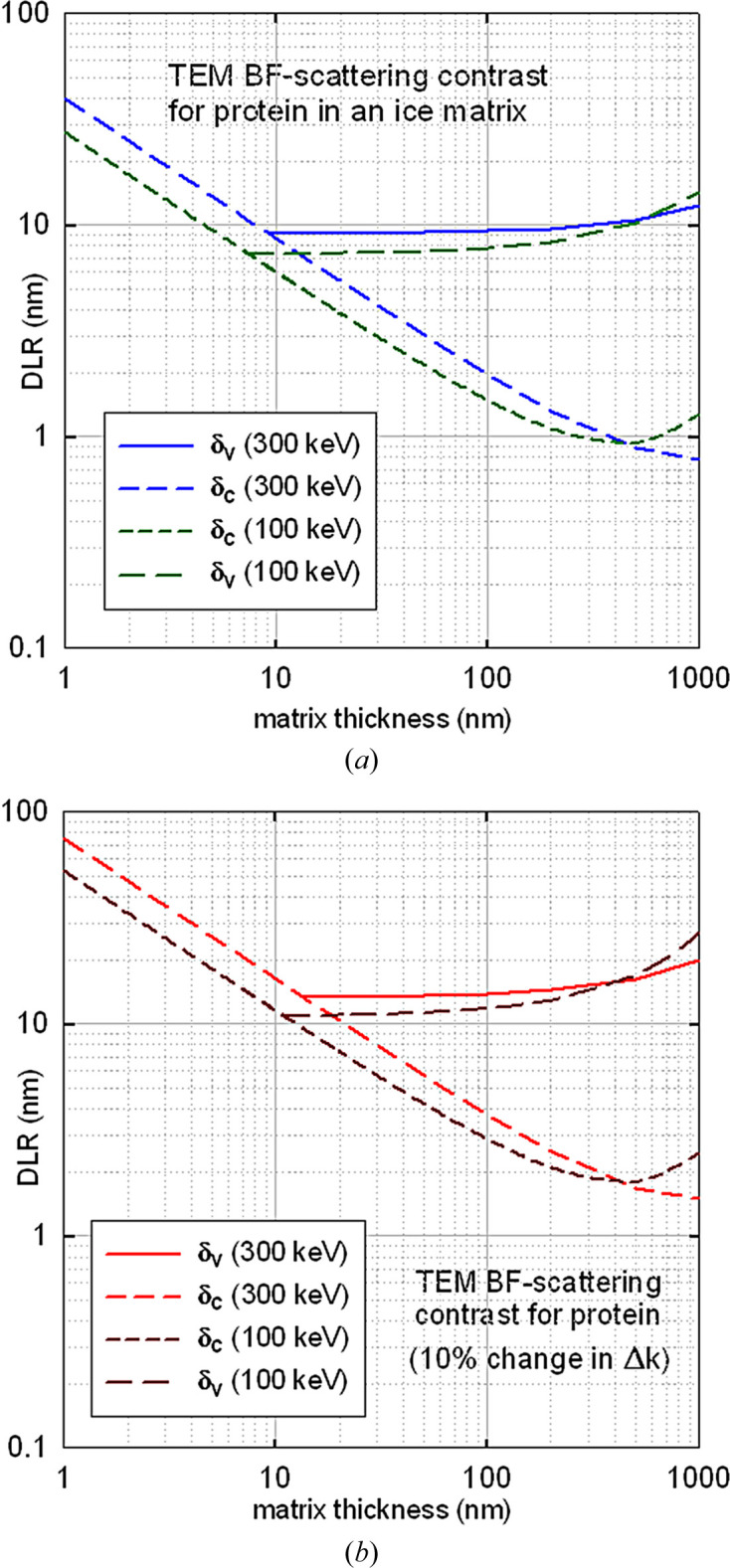
DLR for TEM bright-field electron-scattering contrast with a 5 mrad objective aperture for 100 and 300 keV electrons, the critical dose being as specified in Fig. 3 ▸(*b*).

**Figure 8:**
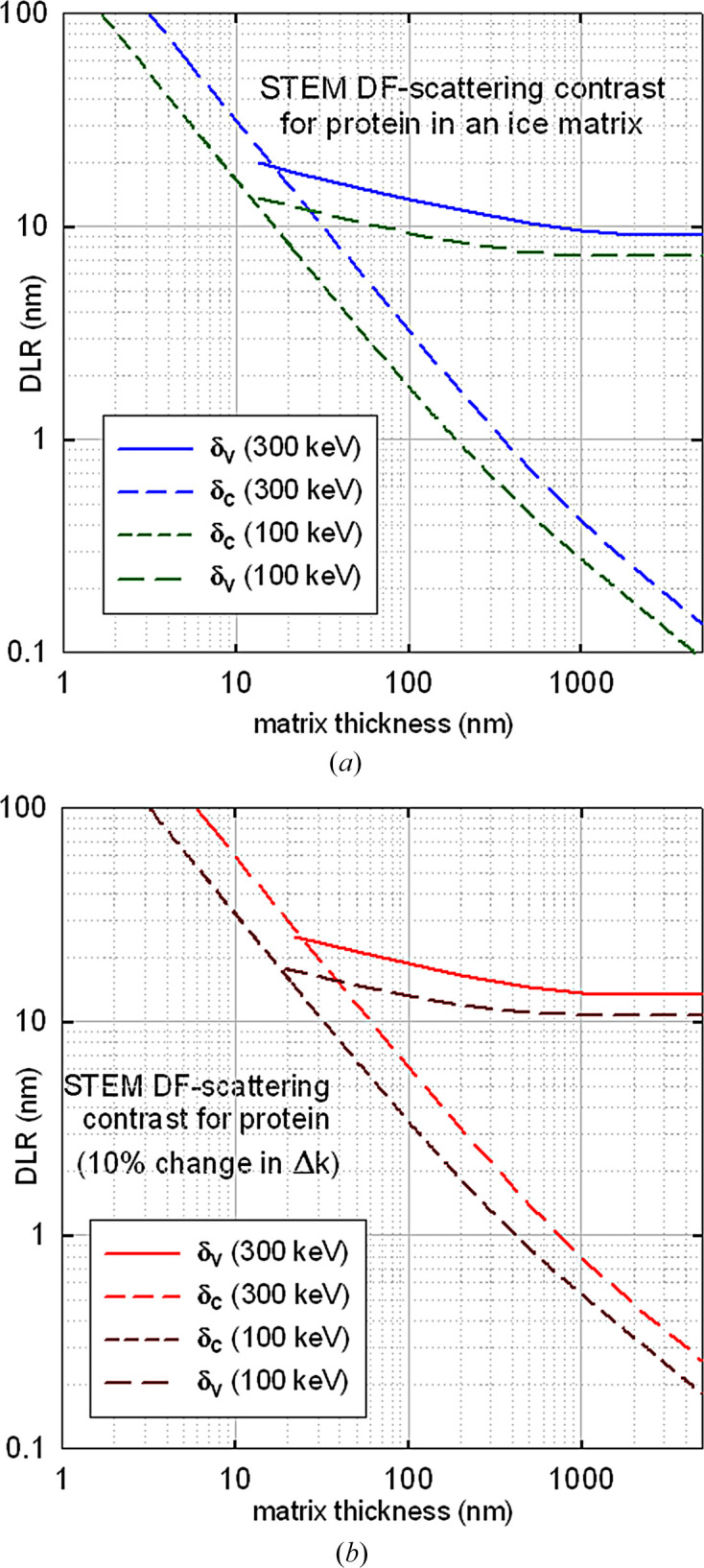
DLR for STEM dark-field electron-scattering contrast (annular-detector inner angle β = 5 mrad) for 100 and 300 keV electrons, the critical dose being as specified in Fig. 3 ▸(*b*).

The analysis given here neglects the possibility of the presence of a substrate or ice layer in the sample. If the layer is not too thick, this situation can be dealt with approximately by including the substrate in the ‘matrix thickness’ (horizonal axis in Figs. 4–8). We also assume that any background due to the X-ray or electron detector is negligible or can be subtracted.

The use of gray units allows a direct comparison between electron and X-ray damage, as well as between data obtained using different electron or photon energies. Even so, we must choose a profile for the resolution-dependent characteristic dose and have based this on the profile shown in Fig. 3 ▸(*b*). For near-atomic resolution (0.2–0.5 nm), *D*_1/e_ is then in the range 20–50 MGy, consistent with the *D*_1/2_ dose limits for cryogenic samples recommended by Henderson (1990 ▸), Owen *et al.* (2006 ▸) and Leal *et al.* (2013 ▸). The characteristic dose will differ for different materials and sample temperatures, but this sensitivity is reduced by the fact that the computed δ_v_ depends only on the quarter or fifth power of *D*_1/e_ or *D*_1/2_.

## 7. X-ray imaging

Organic samples can be examined using soft X-rays in the water window between the photoabsorption edges of carbon (290 eV) and oxygen (540 eV), where low absorption allows the signal-collection efficiency *F* (the ratio of detected to incident photons) to be relatively high. Direct imaging is possible by using a zone plate, with a resolution of typically 30 nm, but this figure may improve in the future, such that the resolution becomes limited by specimen damage. We assume scanned-mode imaging, where the zone-plate efficiency does not further reduce *F*, and take DQE = 0.5 as an estimate of the noise properties of the X-ray detector.

The imaged feature creates a signal that reflects the *difference* in photoelectric absorption power or mean inner potential relative to the ‘matrix’ surroundings. However, X-ray absorption also *attenuates* the signal and reduces the signal efficiency *F*, if defined as the ratio of detected to incident photons. For a columnar feature whose thickness *t* is the same as the surroundings
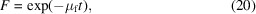
where μ_f_ is the absorption coefficient of the feature. In the case of a feature of height δ (Fig. 1 ▸*e*)
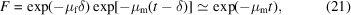
where μ_m_ is the absorption coefficient of the surrounding matrix. The approximation shown in equation (21) is valid if μ_f_ and μ_m_ are close in value, or if δ << *t* (true for most of the data presented here), or if δ << 1/μ_m_ (so that *F* is not much less than 1).

We consider both soft X-rays (500 eV photon energy, wavelength λ = 2.48 nm) and hard X-rays (8 keV energy, λ = 0.155 nm), using the absorption data given in Table 2 ▸.

### 7.1. X-ray absorption contrast

For very thin specimens (*t* << 1/*L*_f_), *F* ≃ exp(−μ_f_*t*) and |*C*| ≃ (μ_f_ – μ_m_)*t*, giving a columnar DLR δ_c_ for specimen features that run from the top to the bottom of the sample. Calculated from equation (10), δ_c_ is represented by the dashed straight lines in Fig. 4 ▸. The voxel DLR δ_v_, depicting the resolution limit for small particles and calculated from equation (11), is initially constant but eventually deteriorates with increasing thickness (as shown by the upward-trending curves) due to the fall in collection efficiency *F* resulting from X-ray absorption. The intersection corresponds to δ_c_ = δ_v_ = *t*, as discussed in Section 3. A sample thickness *t* between 10 and 1000 nm is seen to provide an optimum voxel DLR of about 8 nm (solid blue curve), indicating that protein particles of this size (embedded in ice) should be resolvable for a required dose of about 800 MGy.

Organelles within a biological cell can contain a mixtureof proteins, nucleic acids, lipids and other material, and the complete organelle may appear brighter or darker (positive or negative *C*) with respect to the surrounding material (Nave, 2018 ▸). It may also be possible to distinguish features within a single organelle. To illustrate this, we assume a simple model of an organelle containing proteins with a 10% difference in density, resulting in an about 10% difference in absorption coefficient. The contrast is then (0.1)μ_f_δ and the voxel DLR is about 20 nm at a dose about 1600 MGy. With increasing thickness, δ_v_ degrades faster than for protein in ice, due to the higher absorption coefficient of protein; see Fig. 4 ▸.

For 8 keV X-rays, the absorption coefficients are much smaller and the voxel DLR is calculated to be about 200 nm (for ice thicknesses between 200 nm and 200 µm), making absorption-contrast imaging using hard X-rays uncompetitive with other methods.

### 7.2. X-ray phase contrast

Phase contrast can be obtained by using a Zernike phase plate that subjects the transmitted X-rays (of wavelength λ) to a π/2 phase change. For a weak-phase object of thickness δ, an ideal phase plate produces a contrast given (Rudolph *et al.*, 1990 ▸; Du & Jacobsen, 2018 ▸) by
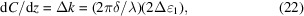
where Δɛ_1_ is the difference in the real part ɛ_1_ of the refractive index (*n* = ɛ_1_ + *i*ɛ_2_) between the imaged feature and its surroundings. The permittivity values shown in Table 3 ▸ result in Δɛ_1_ = 0.49 × 10^−3^ for 500 eV photons (λ = 2.48 nm) and Δɛ_1_ = 1.3 × 10^−6^ for 8 keV photons (λ = 0.155 nm).

Taking *F* from equation (20) and *N*_1/e_ from equation (9), the DLR values given by equations (13) and (14) are shown in Fig. 5 ▸. For protein in ice, the 500 eV voxel DLR (about 6 nm at a fluence of 870 photons nm^−2^) is comparable with the 5 nm resolution estimated by Nave (2020 ▸). Soft X-rays are seen to provide superior DLR for samples thinner than 1 µm, whereas 8 keV photons allow better resolution (15–20 nm) for thicker specimens.

X-ray ptychography using a scanned coherent probe is also an option: Shapiro *et al.* (2014 ▸) reported 5 nm resolution in 2D for a high-contrast radiation-resistant object. In the case of lower contrast features, much worse resolutions are reported: 17 nm for 2D imaging (Deng *et al.*, 2015 ▸) or 45–55 nm for 3D imaging (Deng *et al.*, 2018 ▸) of frozen-hydrated cells, and for 3D imaging of fixed brain 38 nm if stained (Bosch *et al.*, 2023 ▸) or ∼100 nm if unstained (Shahmoradian *et al.*, 2017 ▸).

Hitchcock *et al.* (2024 ▸) have compared spectro-ptychography (SP) with scanning transmission X-ray microscopy (STXM) and concluded that SP is more dose-efficient for scanned areas below 10 µm in size containing strong scatterers. The advantages described for ptychography should also apply for weaker scatterers, provided that the reconstruction algorithms are robust. Examining different holographic and ptychographic methods, Du *et al.* (2020 ▸) showed that photon fluence sets the same limit to the spatial resolution.

## 8. Electron imaging

Thin samples can be examined in the transmission electron microscope (TEM) in conventional (stationary-beam) or in scanning (STEM) mode. The kinetic energy of the electrons, determined by the accelerating voltage, is often in the range 100–300 keV, so we use those two energies in our DLR calculations. Electron optics have improved over the years so that (even *without* an aberration corrector or monochromator) lens aberrations can be relatively unimportant, leaving DLR as the main limitation for beam-sensitive (*e.g.* organic) specimens.

Several image-contrast modes are available, chosen by the detector configuration and electron optics of the TEM column.

### 8.1. Phase-contrast (PC) TEM imaging

Phase contrast is the preferred mode for obtaining high TEM resolution from light-element specimens and is obtained by defocusing the objective lens or by the use of a phase plate in the diffraction plane. Electron holography is also an option (Simon *et al.*, 2008 ▸; Ophus, 2019 ▸; Zhou *et al.*, 2020 ▸).

For the purpose of calculation, we will assume an ideal Zernike phase plate that changes the phase of elastically scattered electrons by π/2 without greatly reducing the detection efficiency *F*. The resulting DLR values will then represent the best-available phase contrast, which might be approximated by a laser phase plate (Schwartz *et al.* (2019 ▸). A hole-free (Volta) phase plate reduces the DQE by about a factor of 2 at a resolution of 0.25 nm (Buijsse *et al.*, 2020 ▸).

For electrons of kinetic energy *E*_0_ and wavelength λ, the phase shift per unit distance *z* within a specimen with mean inner potential Φ is
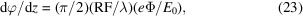
where RF = (1+ *E*_0_/511 keV)/(1+ *E*_0_/1022 keV) is a relativistic factor of 1.09 at *E*_0_ = 100 keV and 1.23 at 300 keV, and *e* is the electron charge. The phase shift is Δφ = (dφ/d*z*)*t* for the columnar situation (Fig. 1 ▸*b*) and Δφ = (dφ/d*z*)δ for a voxel of dimension δ (Fig. 1 ▸*e*). The Weber contrast of a feature is then
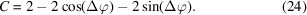


For a weak phase object (Δφ << 1), |*C*_0_| = 2Δφ, giving
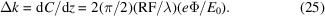


There is evidence that inelastic scattering contributes to phase contrast in thin samples (Kimoto & Matsui, 2003 ▸; Schattschneider & Werner, 2005 ▸; Kabius *et al.*, 2009 ▸; Rose, 2009 ▸; Dickerson & Russo, 2022 ▸), but this may not be so at higher thicknesses (Rez, 2023 ▸). Here, we assume no phase-contrast contribution from mixed scattering, which involves both elastic and inelastic contributions, has a large angular width and predominates in thick samples, leading to severe chromatic broadening. The signal efficiency is then
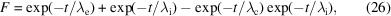
where λ_e_ and λ_i_ are the elastic and inelastic mean free paths for scattering (through all angles) within the appropriate material.

Fig. 6 ▸ shows results for protein in ice and protein (with a 10% difference in MIP) in a protein matrix. Below a sample thickness of 100 nm, voxel DLR values are almost independent of electron energy and are nearly an order of magnitude better than those calculated for X-ray phase contrast. At higher thicknesses, δ_v_ deteriorates due to the reduction in *F*, although less rapidly at higher accelerating voltage. Broadening due to objective-lens chromatic aberration also increases with thickness and could be reduced by energy filtering, *C*_c_ correction or improved lens design.

The predictions shown in Fig. 6 ▸(*b*) are consistent with an estimated resolution of 8.5 nm in 300 keV cryoelectron tomography phase-contrast images of nuclear pore complexes recorded at an objective-lens defocus of 15 µm (Beck *et al.*, 2004 ▸). Blum *et al.* (2026 ▸) have reported 300 kV images of frozen-hydrated apoferritin (defocus 0.6–2.4 µm) at a dose of 100 MGy and with an estimated resolution of 0.4 nm, but using SPA techniques that averaged over 6488 particles.

### 8.2. Bright-field (BF) scattering-contrast TEM

Scattering contrast (also known as diffraction or amplitude contrast) is obtained by inserting a small aperture in a diffraction plane in the TEM or STEM. An on-axis aperture selecting for low scattering angles gives a bright-field image due to variations in (mainly) elastic scattering within the sample (mass–thickness contrast). An aperture whose collection semi-angle β is a few milliradians limits chromatic aberration and optimizes the contrast and DLR (Egerton, 2024 ▸).

Below a certain specimen thickness *t*_max_, the fraction of transmitted electrons passing through an aperture of semi-angle β can be calculated by assuming a Beer’s law expression,
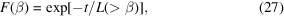
where *L* is a β-dependent scattering length that approximates to the elastic mean free path for scattering through angles *greater* than β (Egerton, 2025 ▸). Values of *t*_max_ have been measured (as a function of β) for a variety of materials and range from 750 nm for amorphous carbon to 150 nm for gold at an electron energy of 300 keV (Hayashida & Malac, 2022 ▸). For thicker samples, Monte Carlo calculations or multiple-scattering theory (Lenz, 1954 ▸) should be used to calculate *F*(β).

Fig. 7 ▸ shows the DLR given by equations (10) and (11), with *F* given by equation (27) and
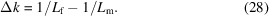
Here, *L*_f_ and *L*_m_ are the Beer’s law parameters for the feature and for the surrounding matrix, calculated using the Lentz–Wentzel atomic model (Lenz, 1954 ▸). The columnar contrast is (Δ*k*)*t* and the voxel contrast is *C* = (Δ*k*)δ.

The voxel DLR deteriorates with increasing thickness because plural scattering causes a reduction in the collection efficiency *F*. However, δ_v_ appears to be slightly lower at 100 keV than at 300 keV. This result is consistent with a preference for 100 keV based on a thin-specimen ‘information coefficient’ for single-particle cryoEM (discussed in Section 9) that allows for chromatic aberration (Peet *et al.*, 2019 ▸). Clearly, 300 keV will be preferable for thick specimens, in which the effect of plural scattering is more severe.

We are neglecting here the possibility of using an electron spectrometer to increase the image contrast *C*. Zero-loss filtering of a bright-field image reduces the collection efficiency *F* by a factor of exp(*t*/λ_i_): about 50 for hydrated tissue of thickness *t* = 1 µm. According to equation (6), the contrast will need to increase by a factor of exp(0.5*t*/λ_i_) ≃ 7 to give an improvement in DLR. Energy filtering also reduces chromatic-aberration blurring of the image, but this effect is small for a small objective aperture.

### 8.3. Dark-field (DF) scattering-contrast STEM

Efficient dark-field imaging is possible in a scanning TEM by using an annular detector that collects almost all of the electrons scattered beyond an angle β, determined by the inner radius of the detector and optical conditions downstream of the specimen. The result is scattering contrast that is complementary to bright-field TEM, the collection efficiency being
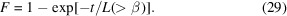
Fig. 8 ▸ shows DLR values given by equations (10) and (11), with *F* taken from equation (29) and Δ*k* from equation (28).

In terms of DLR, dark-field STEM appears comparable with BF scattering contrast for examining thicker specimens. However, beam broadening is also a limiting factor when electrons scattered to high angles are admitted to the detector (Rez *et al.*, 2016 ▸). So, in practice, bright-field STEM imaging is generally preferable for very thick specimens (Hyun *et al.*, 2008 ▸; Sousa *et al.*, 2009 ▸; Egerton, 2025 ▸).

## 9. Comparisons

Du & Jacobsen (2018 ▸) have compared X-ray and electron microscopy of thick hydrated organic specimens, finding that when using 10 keV X-rays, less dose is required for ice thicknesses above 1 µm. Rez (2021 ▸) compared imaging of biological specimens with 200 keV electrons with imaging using soft and hard X-rays. Electrons were found to give superior structural information when elastic scattering is utilized, although soft X-rays in the water window appeared highly competitive. These conclusions are compatible with the DLR results presented here.

Rez *et al.* (2025 ▸) used multislice and Monte Carlo calculations to simulate electron microscopy of thick biological specimens, concluding that noise limits the features that can be distinguished under low-dose conditions. STEM was found to be advantageous (compared with stationary-beam TEM) for both amplitude and phase contrast, with the optimum contrast requiring an accelerating voltage of 700 kV.

Peet *et al.* (2019 ▸) have evaluated the effect of TEM accelerating voltage on phase-contrast images (with particular relevance to SPA) in terms of an information coefficient:
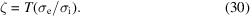
Here, *T* is the fraction of electrons that pass through the specimen without being scattered elastically (cross section σ_e_) or inelastically (cross section σ_i_), the assumption being that both scattering processes inhibit phase contrast because of objective-lens chromatic aberration.

We suggest an alternative figure of merit ζ_0_ for TEM or STEM imaging (including scattering-contrast imaging of thicker specimens) where radiation damage limits the resolution. From equation (6),[Disp-formula fd6]
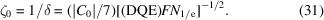
Based on this criterion and Figs. 7 ▸ and 8 ▸, an accelerating voltage of 300 kV offers somewhat better resolution than 100 kV, while for phase contrast there is little difference for thin specimens.

## 10. Discussion

This study has addressed the effect of radiation damage on the resolution of *single* features in a specimen, whose structures may differ from each other. As is well known, greatly improved spatial resolution is possible when there are many identical or near-identical copies of a structure. One example is single-particle analysis (SPA), a cryo-TEM technique used to derive the structure of biological macromolecules (Frank, 2006 ▸). At near-atomic resolution, the signal to noise improves *roughly* as the square root of the number of copies, which for SPA can be many thousands, so that even in the presence of other limiting factors (Rosenthal & Henderson, 2003 ▸) near-atomic resolution becomes achievable.

In the case of molecular crystals the number of molecules can be even larger and direct or diffractive imaging by electrons or X-rays allows atomic-scale resolution, even for very sensitive molecules. The repetitive nature of a crystal means that wave amplitudes rather than intensities are added, an advantage that has been termed a Bragg boost (Spence, 2017 ▸). This advantage derives from the fact that crystallinity concentrates the elastic scattering into narrow angular ranges; see Appendix *A*[App appa].

We have based our analysis on the Rose criterion, which can be justified in terms of the probability of false-positive or false-negative identification (Trebbia, 1988 ▸; Nave, 2020 ▸). However, denoising algorithms (see, for example, Crozier *et al.*, 2025 ▸) can reduce the SNR by as much as a factor of 5 through the use of prior knowledge or by exploiting the correlation of signal between image pixels (and assuming the noise to be uncorrelated). For a recent example, see Wang *et al.* (2026 ▸).

**Figure 9:**
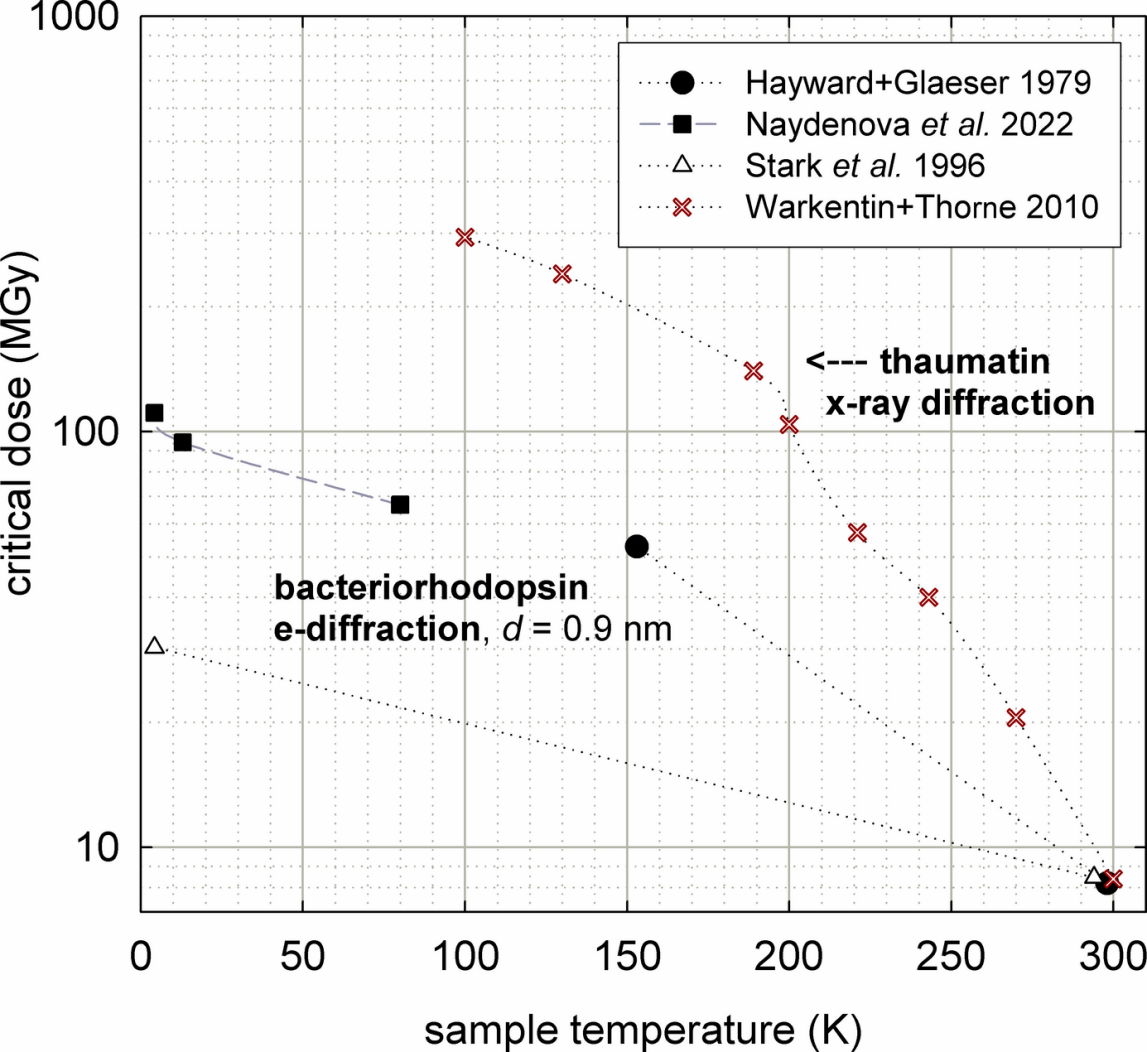
Temperature dependence of the critical (or characteristic) dose *D*_1/e_ for diffraction-pattern fading in bacteriorhodopsin (TEM measurements) and thaumatin (X-ray measurements). The similar values at room temperature are likely fortuitous, since they refer to different materials.

We have taken the radiation-dose limit imposed by radio­lysis to be the same (when expressed in gray units) for electrons and X-rays. Indeed, measurements of room-temperature critical dose for destruction of the carbonyl group in PET gave 460 ± 50 MGy for 320 eV X-rays and 380 ± 50 MGy for 200 keV electrons (Hitchcock *et al.*, 2008 ▸). However, X-ray global damage (based on X-ray diffraction) is often *more* temperature dependent than that caused by electrons, as illustrated in Fig. 9 ▸. The characteristic dose can then be identical (for the same material) only at a single temperature. That temperature seems more likely to correspond to cryogenic conditions, where the temperature-dependent chemical effects of damage are less.

The data shown in Fig. 9 ▸ might suggest that it is always better to cool the sample to the lowest possible temperature. However, TEM specimen charging, bubbling effects and increased specimen drift have been observed at liquid-helium temperature (4 K), and 100 K has been judged to be preferable for single-particle imaging and intermediate temperatures (for example 25 or 42 K) best for lower resolution (2–6 nm) TEM of proteins (Bammes *et al.*, 2010 ▸).

More recently, Dickerson & Russo (2025 ▸) and Dickerson *et al.* (2025 ▸) have shown that specimen movement can be limited by using appropriate support grids and that a temperature of 13 K provides superior resolution. Warkentin & Thorne (2010 ▸) have argued that for the thicker crystals involved in X-ray crystallography, protein diffusion is largely suppressed even at 220 K (above the glass-transition temperature) and that 100 K represents a best choice for collecting structural data sets.

The temperature dependence of later-stage (chemical) damage is likely to be associated with the diffusion of radiation-induced reactive species within the specimen. This diffusion can continue after the irradiation is stopped, leading to the ‘dark progression’ over a period of minutes or hours that has been observed for X-ray radiolysis at sample temperatures above 180 K (Warkentin *et al.*, 2011 ▸). Another phenomenon associated with diffusion is a dose-rate dependence of global damage: the room-temperature characteristic dose increases by a factor of a two or more at high dose rates (Southworth-Davies *et al.*, 2007 ▸; Warkentin *et al.*, 2012 ▸).

Similar dose-rate effects have been observed for electron irradiation, but on a shorter time scale, perhaps arising from the fact that TEM samples are usually less than 1 µm in thickness and the diffusion time is correspondingly short: perhaps proportional to (thickness)^1/2^. However, attempts to model chemical damage are complicated by the fact that diffusion within the irradiated volume is likely to be radiation-enhanced (Egerton, 2019 ▸).

**Figure 10:**
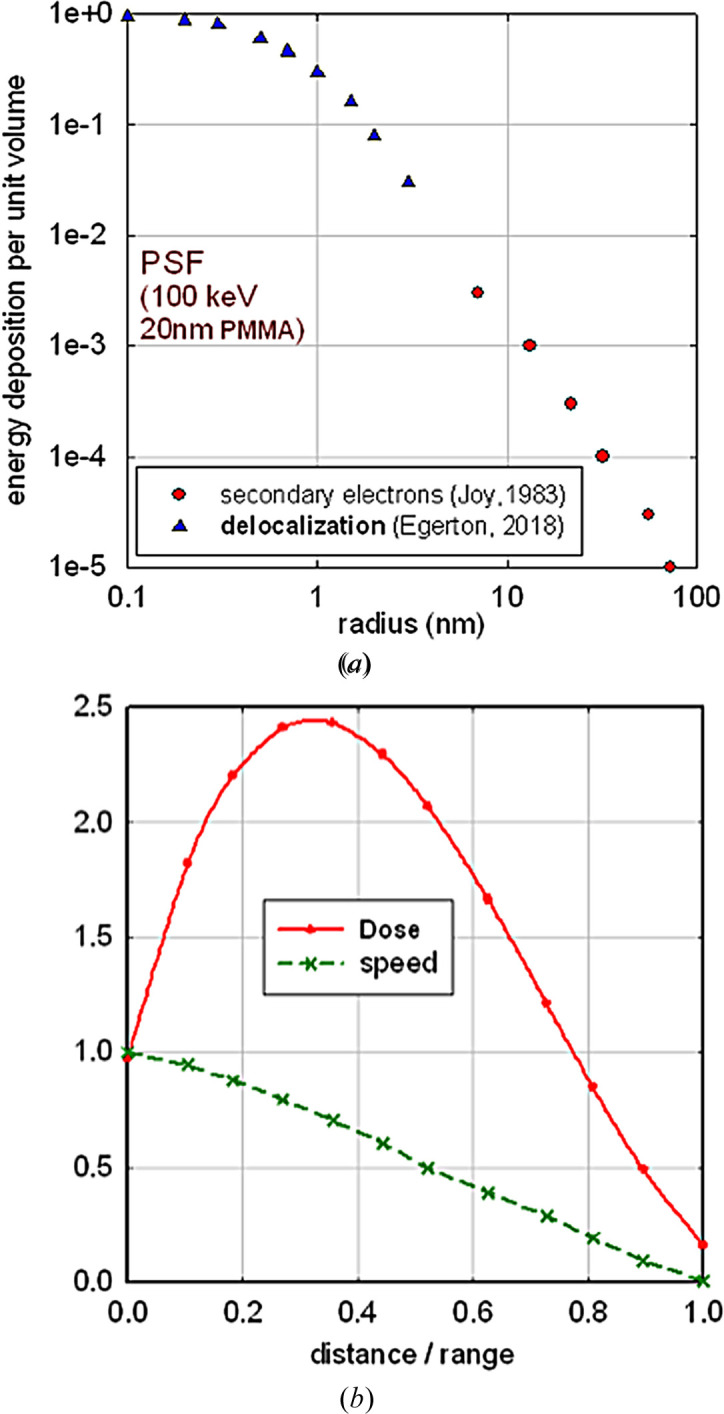
(*a*) Point-spread function for delocalization of electron-beam damage in a 20 nm film of PMMA, estimated from calculations of Coulomb delocalization (Egerton, 2018 ▸) and Monte Carlo calculations of the secondary-electron range (Joy, 1983 ▸). (*b*) Energy deposition by Auger or photoelectrons (following X-ray absorption), as deduced from Monte Carlo calculations (Everhart & Hoff, 1971 ▸), and a derived speed–distance curve that can be used to place a time scale on the energy deposition. Both curves are normalized to 1 at the starting point of the excited electron.

Electron and X-ray damage also differ at the *primary* stage of radiolysis, in terms of the localization of the initial energy deposition. Electron-induced radiolysis begins with the inelastic scattering of primary electrons, which is Coulomb-delocalized on a nanometre scale, but subsequent generation of secondary electrons increases the volume in which energy is deposited. For organic materials, about 50% of the energy is deposited within a radius of 1 nm; see Fig. 10 ▸(*a*).

X-ray radiolysis involves photoabsorption, followed by emission of an electron whose range (for an organic material) can be estimated as *R* (nm) = 0.01*E*^1.3^, where *E* is the electron energy in eV (Reimer, 1998 ▸). Monte Carlo calculations provide a formula (Everhart & Hoff, 1971 ▸) that can be used to predict the *radial dependence* of energy deposition, as depicted in Fig. 10 ▸(*b*). For 500 eV photons, Auger emission generates electrons that deposit half of their energy within 20 nm (and within about 2 fs), while 8 keV X-rays produce high-energy photoelectrons that deposit half of their energy (in 20 fs) within 800 nm. These values exceed our estimated DLR, implying an inaccuracy that could be significant if the feature and matrix densities are substantially different. Near the end of their range, photoelectrons may create high-reaction zones (blobs), which have not been observed in thin TEM specimens.

The localization of radiolysis is relevant to radiation lithography and its time scale is pertinent to the damage caused by pulsed beams. XFEL femtosecond X-ray pulses have provided useful diffraction information prior to damage, but the use of a pulsed electron beam to mitigate damage has been a controversial topic (Egerton, 2025 ▸).

## 11. Summary

We have shown how the concept of damage-limited resolution (DLR) can be applied to both X-ray and transmission electron microscopy of specimens that are beam-sensitive due to radiolytic damage. DLR has an optimum value (δ_opt_, typically of the order of 10 nm) at a specimen thickness *equal* to δ_opt_. Approximate values calculated in this study are given in Table 4 ▸. However, the DLR remains largely unaltered up to a thickness 100 times larger. Beyond that, the damage-limited resolution deteriorates, becoming significantly degraded (except if using hard X-rays) at a thickness of 1 µm.

For electron microscopy, the dose-limited resolution shown in Table 4 ▸ is generally worse than the spatial resolution set by instrumental factors (spherical or chromatic aberration, beam broadening or depth of field), in which case the DLR value will set the resolution limit. For X-ray microscopy, DLR values are mostly better than the resolution provided by the X-ray optics, making instrumental factors dominant.

In previous publications, comparison between hard and soft X-rays, and between electrons and X-rays, has been formulated in terms of the dose required to identify a feature of known size. Here, we address the issue in terms of a damage-limited resolution, allowing for variation of the characteristic dose with feature size. Our general conclusions are that electrons give better resolution than X-rays for thin (<1 µm) samples, while soft X-rays (<520 eV) perform better than harder X-rays (>4 keV) for sample thicknesses below 10 µm. Phase-contrast imaging using hard X-rays is clearly a method of choice for very thick specimens.

When comparing with published image resolutions, it should be remembered that some discrepancy can arise from the choice of Rose criterion (SNR = 3 or 5), the definition of critical fluence (*e.g. N*_1/e_ or *N*_1/2_) and the assumed value of the detector DQE, as well as the choice of solvent matrix (ice, cytosol *etc*.) and the organic (*e.g.* protein) composition. Also relevant is whether the dose is assumed to be absorbed by the imaged feature or whether the analysis includes energy absorption in the surrounding solvent.

Comparison between calculated and experimental resolutions raises a different problem. DLR is based on the total number of particles within sharply constrained boundaries, whereas measurements are based on whatever method is feasible: full-width at half-maximum (FWHM) of a small particle, width of a sharp boundary, contrast transfer function *etc*. These different definitions can give rise to substantially different numbers. Comparison of DLR with instrumental broadening faces the same problem: instrumental effects may be specified as an FWHM or the diameter containing a certain percentage of the electrons. Use of a point-spread function (PSF) removes some of the ambiguity (Egerton & Watanabe, 2022 ▸), but DLR cannot be specified in terms of a PSF.

Expressed in gray units, the characteristic dose for damage has a similar value for electrons and X-rays but is likely not identical, in view of the different temperature dependences involved. The primary (physical) stage of radiolysis is less localized for X-rays, due to the relatively high energies of the Auger electrons and photoelectrons that generate most of the damage. The extent of the subsequent (chemical) damage may also differ: TEM samples are relatively thin, allowing mass loss on a shorter time scale.

## Figures and Tables

**Table 1:** Conversion factors from particle fluence (photons or electrons per nm^2^) to dose in MGy

Material	ρ (g cm^−3^)	500 eV photons	8 keV photons	100 keV electrons	300 keV electrons
Protein	1.35	0.115	0.00102	0.063	0.036
Ice	0.94	0.0097	0.00130	0.065	0.037
PMMA	1.19	0.069	0.00081	0.064	0.037
Carbon	1.8	0.109	0.00056	0.052	0.030

**Table 2:** Absorption length *L* (Henke *et al.*, 1993 ▸) and absorption coefficient μ for some organic materials at X-ray energies of 500 and 8 keV

		500 eV		8 keV	
Material	ρ (g cm^−3^)	*L* (µm)	μ (nm^−1^)	*L* (µm)	μ (nm^−1^)
Protein	1.35	0.67	1.49 × 10^−3^	745	1.34 × 10^−6^
Ice	0.94	8.77	0.114 × 10^−3^	1053	0.95 × 10^−6^
PMMA	1.19	0.97	1.03 × 10^−3^	1340	0.75 × 10^−6^
Carbon	1.8	0.406	1.94 × 10^−3^	1270	0.79 × 10^−6^

**Table 3:** Dielectric data taken from https://henke.lbl.gov/optical_constants/getdb2.html

Material	ρ (g cm^−3^)	1 + ɛ_1_ (500 eV)	1 + ɛ_1_ (8 keV)
Protein	1.35	1.065 × 10^−3^	4.695 × 10^−6^
Ice	0.94	0.578 × 10^−3^	3.403 × 10^−6^

**Table 4:** Calculated damage-limited resolution (in nanometres) for X-ray and electron microscopy of a beam-sensitive sample at a specimen thickness equal to the DLR value and for a dose (in MGy) equal to 100 times the DLR value DLR is tabulated for various contrast mechanisms and for two situations: a protein feature surrounded by ice and a protein feature whose density differs by 10% from that of its protein surroundings.

Contrast mechanism	Energy (eV)	Protein in ice	Protein (10%)
X-absorption	500	8	20
X-ray phase	500	6	22
	8	14	20
TEM phase	100	1.4	5
TEM bright-field	100	7	10
	300	9	13
STEM dark-field	100	13	18
	300	20	25

## Appendix A

As described by Spence (2017 ▸), diffraction from a crystal provides ‘coherent amplification’ of the intensity at each Bragg peak, because wave amplitudes rather than intensities are additive. This argument explains why XFEL atomic-resolution imaging has been achieved using microcrystals but not from noncrystalline particles such as viruses. For a crystal with only ten molecules on each side, crystallinity increases the intensity by a factor of 10^6^, whereas the same number of atoms randomly arranged would provide a scattered intensity only 10^3^ times that of a single molecule.

We point out here that the ‘Bragg boost’ is achieved through a redistribution of the scattered intensity, rather than any increase in the total. In other words, the crystal does not act like a resonating system, extracting more energy from the incident beam. The factor of 10^6^ above refers to the peak intensity at a Bragg peak, not to the intensity integrated over the peak. In terms of our DLR analysis, crystallinity may increase the image contrast *C* but not the signal efficiency *F*.

The limited size of a crystal broadens the reciprocal-lattice ‘point’ in all three dimensions, even if the crystal is otherwise perfect. This broadening leads to enlarged spots on the detector while, in the beam direction, it leads to an increase in the angular range over which the reflection occurs. For an isotropic-shaped crystal, the broadening is proportional to 1/*L*, where *L* is the dimension of the crystal. If *a* is the lattice spacing, the number of unit cells *n* in each direction is equal to *L*/*a* and the separation between Bragg peaks is proportional to 1/*a*. The proportion of the volume occupied by the Bragg peaks in reciprocal space is therefore *n*^−3^, reducing the increase in the *integrated* intensity to a factor of 10^3^ (rather than 10^6^) in the example above.

However, sharpening of the Bragg peaks makes it easier to distinguish them from the background in a diffraction pattern. Where diffractive imaging is used to derive electron phase and construct an image of the specimen, this advantage may be decisive.

The above arguments apply only under conditions of kinematic diffraction, where the crystal dimensions are small compared with the extinction distance of the X-rays or electrons. If the diffraction is dynamical, intensity can be scattered back into the central (‘undiffracted’) beam, leading to primary extinction. A crystalline feature could then provide *less* contrast than a same-sized amorphous feature if the imaging makes use of integrated intensity, as in bright- or dark-field electron microscopy.
